# Mixotrophic cyanobacteria are critical active diazotrophs in polychlorinated biphenyl-contaminated paddy soil

**DOI:** 10.1093/ismeco/ycae160

**Published:** 2025-03-18

**Authors:** Wenbo Hu, Ying Teng, Xiaomi Wang, Yongfeng Xu, Yi Sun, Hongzhe Wang, Yanning Li, Shixiang Dai, Ming Zhong, Yongming Luo

**Affiliations:** Key Laboratory of Soil and Sustainable Agriculture, Institute of Soil Science, Chinese Academy of Sciences, Nanjing 211135, China; College of Resources and Environment, University of Chinese Academy of Sciences, Beijing 100049, China; College of Resources, Environment and Earth Science, University of Chinese Academy of Sciences, Nanjing 211135, China; Key Laboratory of Soil and Sustainable Agriculture, Institute of Soil Science, Chinese Academy of Sciences, Nanjing 211135, China; College of Resources, Environment and Earth Science, University of Chinese Academy of Sciences, Nanjing 211135, China; Key Laboratory of Soil and Sustainable Agriculture, Institute of Soil Science, Chinese Academy of Sciences, Nanjing 211135, China; College of Resources, Environment and Earth Science, University of Chinese Academy of Sciences, Nanjing 211135, China; Key Laboratory of Soil and Sustainable Agriculture, Institute of Soil Science, Chinese Academy of Sciences, Nanjing 211135, China; College of Resources, Environment and Earth Science, University of Chinese Academy of Sciences, Nanjing 211135, China; Key Laboratory of Soil and Sustainable Agriculture, Institute of Soil Science, Chinese Academy of Sciences, Nanjing 211135, China; College of Resources and Environment, University of Chinese Academy of Sciences, Beijing 100049, China; College of Resources, Environment and Earth Science, University of Chinese Academy of Sciences, Nanjing 211135, China; Key Laboratory of Soil and Sustainable Agriculture, Institute of Soil Science, Chinese Academy of Sciences, Nanjing 211135, China; College of Resources and Environment, University of Chinese Academy of Sciences, Beijing 100049, China; College of Resources, Environment and Earth Science, University of Chinese Academy of Sciences, Nanjing 211135, China; Key Laboratory of Soil and Sustainable Agriculture, Institute of Soil Science, Chinese Academy of Sciences, Nanjing 211135, China; College of Resources and Environment, University of Chinese Academy of Sciences, Beijing 100049, China; College of Resources, Environment and Earth Science, University of Chinese Academy of Sciences, Nanjing 211135, China; Key Laboratory of Soil and Sustainable Agriculture, Institute of Soil Science, Chinese Academy of Sciences, Nanjing 211135, China; College of Resources, Environment and Earth Science, University of Chinese Academy of Sciences, Nanjing 211135, China; Key Laboratory of Soil and Sustainable Agriculture, Institute of Soil Science, Chinese Academy of Sciences, Nanjing 211135, China; Key Laboratory of Soil and Sustainable Agriculture, Institute of Soil Science, Chinese Academy of Sciences, Nanjing 211135, China; College of Resources, Environment and Earth Science, University of Chinese Academy of Sciences, Nanjing 211135, China

**Keywords:** active diazotrophs, cyanobacteria, PCB contamination, paddy soil

## Abstract

Biological nitrogen fixation by diazotrophs is a crucial biogeochemical process in global terrestrial ecosystems, especially in nitrogen-limited, organic-contaminated soils. The metabolic activities of diazotrophs and their ability to supply fixed nitrogen may facilitate the transformation of organic pollutants. However, the active diazotrophic communities in organic-contaminated soils and their potential metabolic functions have received little attention. In the current study, the relationship between biological nitrogen fixation and polychlorinated biphenyl (PCB) metabolism was analyzed *in situ* in paddy soil contaminated with a representative tetrachlorobiphenyl (PCB52). ^15^N-DNA stable isotope probing was combined with high-throughput sequencing to identify active diazotrophs, which were distributed in 14 phyla, predominantly *Cyanobacteria* (23.40%). Subsequent metagenome binning and functional gene mining revealed that some mixotrophic cyanobacteria (e.g. FACHB-36 and *Cylindrospermum*) contain essential genes for nitrogen fixation, PCB metabolism, and photosynthesis. The bifunctionality of *Cylindrospermum* sp. in nitrogen fixation and PCB metabolism was further confirmed by metabolite analyses of *Cylindrospermum* sp. from a culture collection as a representative species, which showed that *Cylindrospermum* sp. metabolized PCB and produced 2-chlorobiphenyl and 2,5-dihydroxybenzonic acid. Collectively, these findings indicate that active diazotrophs, particularly mixotrophic cyanobacteria, have important ecological remediation functions and are a promising nature-based *in situ* remediation solution for organic-contaminated environments.

## Introduction

Unregulated dismantling of electronic waste (e-waste) and industrial thermal process by-products are now the primary sources of polychlorinated biphenyl (PCB) contamination [1, 2]. PCB metabolism in soils is challenging and the process may be limited by available nitrogen [3, 4]. Direct supplementation with nitrogen has been shown to promote the degradation of polycyclic aromatic hydrocarbons (PAHs), polybrominated diphenyl ethers, and PCBs in soils and constructed wetlands [4–6]. However, the low utilization efficiency of nitrogen fertilizer may trigger other environmental issues, such as nonpoint source pollution and potential inhibition of pollutant degradation due to excessive production of intermediate metabolites [7, 8].

An alternative to nitrogen supplementation is biological nitrogen fixation (BNF) by diazotrophs. BNF converts atmospheric nitrogen into bioavailable ammonia, which is the primary source of bioavailable nitrogen in soils [9, 10]. BNF is crucial for maintaining global ecosystem primary productivity and biogeochemical cycling of elements [11, 12]. In nitrogen-deficient, organic-polluted soils, BNF can increase bioavailable nitrogen content, promoting the growth of other microorganisms, including those capable of degrading organic pollutants [13–15]. For instance, the nitrogen fixer *Azotobacter chroococcum* HN can provide nitrogen to the degrader *Paracoccus aminovorans* HPD-2 by forming “bridge structures” that facilitate the co-metabolic degradation of pyrene [14]. In soil polluted with crude oil, seeding with adapted *Azotobacter* increased the crude oil degradation rate by 9%–10%, and introducing autochthonous diazotrophs to existing degrader communities improved diesel degradation rates by 3.4%–6.9% [13, 16].

In addition to fixing nitrogen, some diazotrophs have PCB detoxification genes that enable them to reduce environmental contamination through aerobic degradation and anaerobic dechlorination pathways. These bifunctional microorganisms highlight the significant ecological function of the diazotrophic community in polluted sites [7, 17]. For example, the aerobic diazotroph *Burkholderia xenovorans* LB400 exhibits a 2, 4′-chlorobiphenyl degradation rate of 85% [18]. Some anaerobic diazotrophs, such as *Dehalococcoides mccartyi*, also catalyze the reductive dechlorination of 2,3,4,5,6-pentachlorobiphenyl [19–21]. Additionally, the hydrogen gas produced as a byproduct of nitrogen fixation by these diazotrophs can act as an electron donor, thereby facilitating the reductive dechlorination of PCBs [22, 23]. However, our understanding of which diazotrophs are active *in situ* and their metabolic potential in organic-polluted soils remains limited.

Paddy soils provide a source of food for nearly half of the world’s population but have higher PCB loads than other soil use types [24, 25]. The unique cyclical drought–flood management pattern of these soils stimulates the nitrogenase activity of various microorganisms [26]. Cyanobacteria are the primary contributors to BNF in rice–soil systems. These oxygenic photosynthetic prokaryotes are among the planet’s earliest organisms and contributed significantly to the evolution of Earth’s atmosphere and the biogeochemical cycles of carbon and nitrogen [26–28]. In this study, we examine whether cyanobacteria have the ability to metabolize organic pollutants while performing BNF in organic-contaminated paddy soils. As the study context, a typical paddy soil contaminated with PCBs from Taizhou, China, was selected. This region has a long history of e-waste dismantling spanning 40 years [2, 29]. We selected PCB52 (2,2′,5,5′-Tetrachlorobiphenyl), a typical local pollutant, as a model PCB and combined ^15^N-DNA stable isotope probing (SIP) with metagenomic analysis to (i) investigate the active diazotrophic communities and assess their phylogeny and metabolic potential and (ii) study the nitrogen-fixing ability and PCB metabolism mechanisms of select representative strains of active diazotrophs.

## Materials and methods

The experimental design of this study mainly included physicochemical analysis, nitrogen fixation index analysis, DNA-SIP, Illumina MiSeq sequencing, metagenomic sequencing, and functional gene mining. The schematic diagram of the experimental workflow is shown in Fig. S1, and the detailed operations are described below.

### Sample collection and soil microcosm incubation

Surface soil (0-20 cm depth) was collected from a paddy field in Luqiao, Taizhou City, Zhejiang Province, China (28°32′ 24′′N, 121°21′36′′ E). The sampled site is an abandoned long-term e-waste disposal site, and the range of PCB concentrations in the soil is ~0.016–1.0 mg kg^−1^ [30]. The soil samples were transported to the laboratory at low temperature. After removing visible plant tissues, gravel, and debris, the samples were mixed thoroughly and divided into two parts. One part was stored at 4°C for use in soil microcosm experiments, and the other part was air dried and used to determine the basic physicochemical properties of soil (Table S1, Text S1).

Soil microcosms were prepared by placing 10.0 g of paddy soil in a 100 ml serum bottles (Macherey-Nagel GmbH, Düren, Germany); next 20.0 ml of ultrapure water was added to flood the soil with water to 1 cm above the soil surface [31]. The culture bottles were sealed with sterile sealing film and then placed in an artificial climate chamber for 7 days. The climate chamber was maintained at a light intensity of 150–180 μmol m^−2^ s^−1^, a 16:8 h light:dark cycle, and temperatures of 27°C and 20°C during the light and dark periods, respectively. Emergent weeds were removed while retaining the photoautotrophic biofilm on the water surface. On day 7, 24 μl of a stock solution of PCB52 (99.5% purity; in acetone; Dr Ehrenstorfer, Ausberg, Germany) was added to the serum bottles to obtain a final PCB52 concentration of 1.2 mg kg^−1^. After 2 h of horizontal shaking, the permeable membrane was replaced with a butyl rubber plug and sealed tightly with an aluminum foil lid. To exclude the effect of trace acetone, an equal volume of acetone solvent was added to the control group. In the isotope-labeled group, the air in the headspace was replace with ^15^N-labeled N_2_ (99.0% purity; Sigma-Aldrich, St. Louis, MO, USA) and O_2_ at a ratio of 4:1 by volume to allow the identification of active diazotrophs by the stable isotope method. The bottles were then returned to the original climate chamber for further incubation. To exclude the effect of soil adsorption of PCB52, a sterile control (autoclaved three times at 121°C and 1 h at 24 h intervals) was also set up simultaneously. All treatments had 4 biological replicates, and samples were taken on days 0, 7, 14, 28, 42, and 56 for DNA extraction, analysis of soil nitrogenase activity analysis, and detection of residual PCB52 (Text S2).

### Determination of soil residual polychlorinated biphenyl contents and nitrogenase activity

Soil residual PCB52 was extracted as described previously [32]. Briefly, 2.0 g of freeze-dried soil sample was weighed into a 50 ml clean brown glass centrifuge tube, and 10 ml of n-hexane (high-performance liquid chromatography grade; TEDIA, Ohio, USA) was added. The tubes were vortexed to mix the contents thoroughly and then left in the dark for 12 h for extraction. Next, ultrasonic extraction was performed for 45 min at 25°C and 100% frequency. The supernatant was centrifuged for 5 min at 1500 rpm, and 2.0 ml of the supernatant was passed through a 0.22 μm organic-phase filter membrane (Tapery, Nanjing, China) into a brown injection flask, and then quantified using a GC7890 gas chromatograph (Agilent Technologies, Santa Clara, CA) equipped with an HP5 column (30 m × 0.32 mm × 0.25 μm) by reference to a standard curve (*R*^2^ = 0.9998) constructed using an external standard solution. A quality control sample was examined every 10 samples, and the relative deviation was 89.6%–102.2%.

Soil nitrogenase activity was determined by the acetylene reduction assay [33, 34]. Ten percent of the air in the headspace of the soil microcosm bottles was replaced with high-purity acetylene, and the bottles were incubated in a climate chamber for 24 h. Next, the ethylene content in the headspace was analyzed on a gas chromatograph (Agilent Technologies, Santa Clara, CA) equipped with a hydrogen flame ionization detector (FID) and a Porapak T4 mm × 2 m packed column. The column temperature was 60°C, the injector temperature was 120°C, the FID detector temperature was 220°C, and the carrier gas was high-purity nitrogen.

### Deoxyribonucleic acid extraction and quantitative polymerase chain reaction

Genomic DNA was extracted from ~0.5 g of soil collected from the microcosms on days 0–56 using a FastDNA spin kit for soil (MP Biomedicals, Santa Ana, CA) according to the manufacturer’s instructions. Quantitative PCR was used to quantify the copy number of (i) the 16S ribosomal ribonucleic acid (rRNA) gene to analyze the whole bacterial community (515F/907R), (ii) the *nifH* gene to assess the diazotrophic community (polF/polR) [35], (iii) the *bphC3* gene to evaluate PCB degrading bacteria (bphC-q3-188f/bphC-q3-333r) [36] and (iv) the *Dehalococcoides* (*Dhc*) 16S rRNA gene to evaluate PCB dechlorinating bacteria (C-DehalF/C-1100R) [36]. Details of the methodology are provided in Table S2 and Text S3.

### Stable isotope probing gradient fractionation

Genomic DNA isolated from the ^15^N-labeled soil microcosms on day 56 was separated into “heavy” (i.e., ^15^N-DNA) fractions and “light” (i.e., ^14^N-DNA) fractions by CsCl gradient ultracentrifugation [23, 26]. The concentration of DNA from four parallel extractions was measured with a Qubit 2.0 Fluorimeter (dsDNA high-sensitivity assay kit; Invitrogen, Carlsbad, CA, USA). Approximately 5.0 μg of gDNA was mixed with a CsCl gradient to obtain a buoyant density of 1.725 g mL^−1^ in 4.9 mL OptiSeal polyallomer tubes (Beckman Coulter, Palo Alto, USA). The tubes were centrifuged at 274952 g for 69 h at 20°C in an Optima XPN-100 ultracentrifuge (Beckman Coulter, Palo Alto, USA). The resulting DNA gradients were fractionated into 30 equal volumes (∼150 μl) using an NE-1000 single-syringe pump (New Era Pump Systems, Farmingdale, NY). After measuring the buoyancy density of each fraction using an AR200 digital refractometer (Reichert, Buffalo, NY), the DNA fractions were further purified (Text S4).

### Illumina MiSeq sequencing and analysis

Light and heavy DNA samples were identified based on the results of fluorescence quantification and buoyancy density analysis of each CsCl gradient fraction, followed by high-throughput sequencing analyses of 16S rRNA gene amplicons of ^14^N and ^15^N heavy layer DNA. Amplification obtained using 16S rRNA primers were purified using the AxyPrep DNA Gel Extraction Kit (Axygen Biosciences, Union City, CA, USA) and quantified in a Quantus™ Fluorometer (Promega, USA). Next, libraries were constructed with the NEXTflex™ Rapid DNA-Seq Kit (Bioo Scientific, USA) and sequenced on the Illumina MiSeq PE300 (Majorbio Biopharm Technology Co. Ltd., Shanghai, China). The data were processed as described previously [23]. Briefly, quality control of the raw sequences was performed using fastp software (https://github.com/OpenGene/fastp, version 0.20.0), and sequences were spliced using FLASH (http://www.cbcb.umd.edu/software/flash, version 1.2.7). The spliced sequences were subjected to noise reduction with DADA2 in Qiime2, and the taxonomic information of the resulting amplicon sequence variants (ASVs) was identified based on the Silva 16S rRNA database (v 138). Active diazotrophs were identified using the DESeq2 package in R software [23, 37]. ASVs that were significantly more abundant in the heavy layer DNA of the ^15^N_2_ microcosms than in the heavy layer DNA of the ^14^N_2_ microcosms were identified as labeled taxa, i.e., active diazotrophs (Table S3). Tax4Fun2 was used to predict the functional potential of the active diazotrophic communities [38]. Reference sequences were retrieved from the NCBI GenBank database for phylogenetic tree construction. The 16S rRNA gene sequences were aligned using MUSCLE v5.1 [39], and the best-fit model (bootstrap = 1000) implemented in iQ-TREE v1.6.12 was used to infer the maximum likelihood phylogenetic tree [40]. Finally, the Interactive Tree Of Life (iTOL, https://itol.embl.de/) was used to visualize and annotate the resulting tree [41].

### Metagenomic sequencing and analysis

The small quantity of DNA in a single SIP fraction was inadequate for shotgun metagenomic sequencing. Consequently, a single composite DNA sample was created by pooling the heavy DNA fractions with the greatest target gene abundances in each duplicate ^15^N treatment. The composite DNA was fragmented to a size of ~450 bp using Covaris M220 (Covaris, Woburn, MA) and used to construct paired-end (PE150) sequencing libraries. Sequencing was performed on the Illumina Novaseq 6000 platform (Illumina Inc., San Diego, CA) at Shanghai BIOZERON BioTechnology CO., Ltd. Each sample produced ~10 billion base pairs (~10 Gbp) of sequence reads. The assembly, binning, taxonomic classification, and functional annotation of the raw reads were performed as described by Dong et al. [42] (Text S5). The phylogenetic trees were constructed using the UBCG pipeline [43] (Text S5). The genomic information of all *Cylindrospermum* (a genus was detected in SIP fractions) species was obtained from NCBI GenBank (Table S4), and the genomes of these species were annotated using Eggnog Mapper (http://eggnog-mapper.embl.de/). Collated amino acid sequences of PCB degradation genes from the NCBI database (only *Cyanobacteria*) and the Pfam database (http://pfam.xfam.org/) were used to construct an amino acid database of functional PCB degradation genes (https://github.com/Seraphxiaoxiao/PCB-degradation-functional-genes). The PCB degradation genes of the metagenome-assembled genomes (MAGs) and three genomes of *Cylindrospermum* species were identified by searching the constructed database using Blastp (v2.13.0, e-value <10^−5^) [44].

### *Cylindrospermum* sp. strain, media, microscopy, and nitrogenase activity

*Cylindrospermum* sp. (accession number in culture collection: FACHB-282) was obtained from the Freshwater Algae Culture Collecting of the Institute of Hydrobiology (FACHB) at the National Aquatic Biological Resource Center (Wuhan, China). *Cylindrospermum* sp. is the only strain of *Cylindrospermum* available for purchase from FACHB. The strain was cultured in modified nitrogen-free BG11 medium [45] (Text S6). BG11-C medium in which the sodium carbonate in BG11 medium was replaced with sodium chloride was used for PCB degradation.

Genomic DNA was extracted from log-phase bacterial cultures grown in BG11 medium using a bacterial genomic DNA extraction kit (TaKaRa, Tokyo, Japan). The 16S rRNA gene sequences were analyzed by PCR and Sanger sequencing for taxonomic identification based on the NCBI GenBank database. Detailed steps and the DNA sequence information obtained from sequencing are described in Text S7. Specific procedures for the microscopy and the determination nitrogenase activity of *Cylindrospermum* sp. are detailed in Text S8.

### Growth and polychlorinated biphenyl 52 degradation during *Cylindrospermum* sp*.* cultivation

*Cylindrospermum* sp. was inoculated in fresh BG11-N liquid medium, and the culture was incubated in the climate chamber with shaking at 120 rpm for two weeks until reaching the exponential growth phase. The culture was transferred to a sterile centrifuge tube in a sterile operating environment and centrifuged at 5000 rpm for 15 min. The supernatant was discarded and the cells were washed twice with BG11-C medium. The OD680 of the final suspension was adjusted to 0.38. Meanwhile, 30 μl of 200 mg L^−1^ PCB52 (prepared in acetone) was added to a 50 mL sterile transparent borosilicate glass culture flask containing 5 mL of BG11-C medium (final PCB52 concentration of 1.2 mg L^−1^). After the acetone was completely volatilized, 250 μl of prepared bacterial suspension (OD680 = 0.38) was added to the sealed bottle, which was then incubated in the climate chamber with shaking at 120 rpm. Although the double ortho-position of PCB52 has extremely strong photochemical resistance [46], the photodegradation of PCB52 by ultraviolet light was minimized by using a light-emitting diode light suitable for the cyanobacteria growth and borosilicate glass culture bottles, which filter out most wavelengths of ultraviolet light. A negative control group (Control) with no culture present was performed in the same condition. Destructive sampling was performed on days 0, 3, 6, 9, and 15 to extract chlorophyll a, PCB52, and degradation intermediates (Text S9). Four replicates were set up for each of the culture bottles used for the detection of all indicators at each time point.

### Statistical analysis and data visualization

Statistical analysis was conducted with the R platform (version 4.3.1). Parameters in different groups were compared by Student’s t test for parametric data or the Wilcoxon test for nonparametric data in the “ggsignif” package [47]. Correlations based on the measurements of Spearman coefficients between different parameters were calculated in R and visualized using the “ggpubr”, “dplyr”, and “ggplot2” packages.

## Results

### Nitrogen fixation capacity and polychlorinated biphenyl metabolism are positively correlated in paddy soil

**Figure 1:**
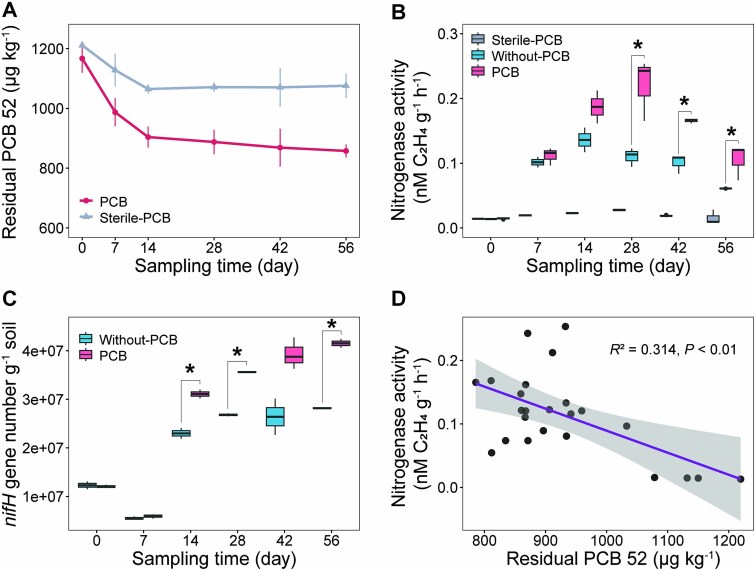
Changes in PCB52 concentration, *nifH* gene abundance, and nitrogenase activity in PCB52-contaminated paddy soil. (A) Dynamics of soil residual PCB52 over a 52-day incubation period. Mean PCB52 concentrations are shown; the vertical lines indicate the standard deviation (SD; *n* = 4 biologically independent bacterial cultures per treatment group). Sterile-PCB: sterile control with PCB addition; PCB: PCB addition. (B and C) Comparison of nitrogenase activity (B) and *nifH* gene abundance (C) between the three treatment groups. The horizontal bars in the box plots represent medians. The 75th and 25th percentiles are represented by the tops and bottoms of the boxes, respectively. Sterile-PCB: sterile control with PCB addition; PCB: PCB addition. Without-PCB: without PCB addition. (D) Correlation between nitrogenase activity and soil residual PCB52. The shaded areas denote the 95% confidence intervals of the regression lines. Significant differences between sample types are indicated by ^*^ (^*^P < .05).

The effects of PCBs on diazotroph abundance and nitrogenase activity in paddy soil were investigated by setting up three different treatments: a PCB52-amended group (PCB), a group without PCB52 addition (Without-PCB), and a sterile control (Sterile-PCB). After 56 days of incubation, the PCB52 removal rate was 17.42% higher in PCB group than that in the Sterile-PCB group, indicating the involvement of microorganisms in PCB52 degradation (Fig. 1). Nitrogenase activity was also significantly higher in the PCB group than in the Without-PCB group at 28 d, 42 d, and 56 d but remained low in the Sterile-PCB group (Fig. 1B). A similar trend was observed in *nifH* gene abundance, which was significantly higher in the PCB group than in the Without-PCB group at 14 d, 28 d, and 56 d (Fig. 1C). Furthermore, there was a significant negative linear correlation between the residual PCB52 concentration and nitrogenase activity (Fig. 1D).

**Figure 2:**
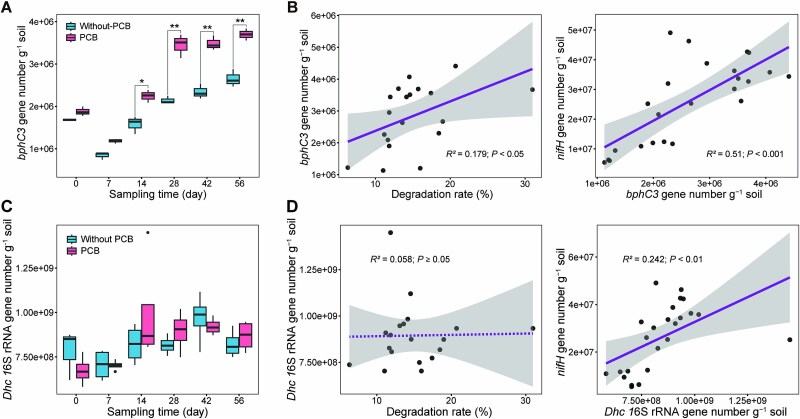
Changes in *bphC3* and *Dhc* 16S rRNA gene abundances in PCB52-contaminated paddy soil. (A and C) Comparison of *bphC3* (A) and *Dhc* 16S rRNA gene (C) abundances between the without-PCB and PCB groups (^*^P < .05, ^*^^*^P < .01). The horizontal bars in the box plots represent medians. The 75th and 25th percentiles are represented by the tops and bottoms of the boxes, respectively. (B) Correlations of *bphC3* gene abundance with the PCB52 degradation rate and *nifH* gene abundance. (D) Correlations of *Dhc* 16S rRNA gene abundance with PCB52 degradation rate and *nifH* gene abundance. PCB52 degradation rate (%) = dissipation rate of the experimental group – adsorption rate of sterilized control group = [(initial concentration – residual concentration)/(sterile initial concentration – sterile residual concentration)] × 100%. The shaded areas denote the 95% confidence intervals of the regression lines.

Because the results in Fig. 1 implicated that microorganisms were involved in PCB52 metabolism, the abundances of *bphC3* (for PCB degrading bacteria) and *Dhc* 16S rRNA gene (for PCB dechlorinating bacteria) were quantified by qPCR. Although both *bphC3* and *Dhc* 16S rRNA gene abundances showed significant positive linear correlations with *nifH* gene abundance (Fig. 2), their abundance patterns differed between treatments and time points. As shown in Fig. 2A, from 14 day, *bphC3* gene abundance was significantly higher in the PCB group than in the Without-PCB group. In addition, the PCB52 degradation rate was positively correlated with *bphC3* gene abundance (Fig. 2A and B). By contrast, *Dhc* 16S rRNA genes abundance did not differ significantly between the PCB and Without-PCB groups at different time points, nor was it correlated with the PCB52 degradation rate (Fig. 2C and D). These results suggest that PCB-degrading bacteria contributed to the positive correlation between the soil PCB dissipation and nitrogenase activity.

### Identification of active diazotrophs in polychlorinated biphenyl-contaminated paddy soil using deoxyribonucleic acid-stable isotope probing

**Figure 3:**
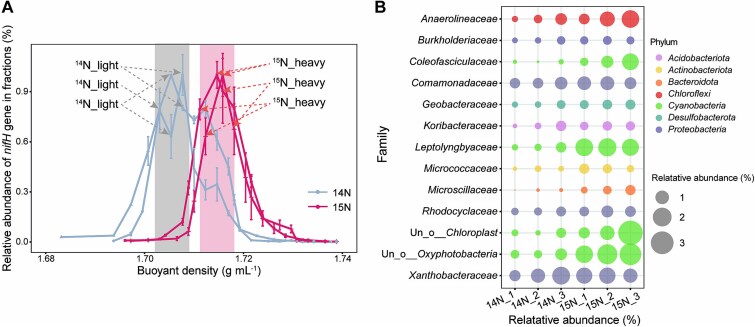
SIP of active diazotrophs. (A) Shift in the relative abundance of the *nifH* gene with changing buoyant density. The mean abundance is shown; the vertical lines represent the SD. The background indicates the heavy and light fractions. (B) Bacterial community composition as represented by the most abundant family in the heavy DNA fractions of the SIP experiments. The size of each circle represents relative abundance, and the circles are categorized at the phylum level.

DNA was extracted from PCB52-amended soil microcosms incubated with ^14^N-N_2_ and ^15^N-N_2_ fractionated by CsCl density gradient ultracentrifugation. The quantification of *nifH* gene copy numbers across the fractions allowed the definition of heavy and light DNA fractions based on *nifH* gene abundance and buoyancy density (Fig. 3A). DESeq2 analysis identified 105 ASVs representing active diazotrophs. These active diazotrophs were distributed across 14 phyla, predominantly *Cyanobacteria* (23.40%) and *Proteobacteria* (16.99%), with the remainder belonging to *Acidobacteria*, *Actinobacteria*, *Armatimonadetes*, *Bacteroidetes*, *Chlorobi*, *Chloroflexi*, *Desulfobacterota*, *Firmicutes*, *Ignavibacteriota*, *Nitrospirae*, *Planctomycetes*, and *Verrucomicrobia* (Fig. S2). Among these, 13 families from 7 phyla had relative abundances of >1% in the heavy fraction (Fig. 3B). The relative abundance of *Cyanobacteria* was significantly higher in the heavy fraction of ^15^N microcosms than in the ^14^N microcosms (Fig. S3). *Cyanobacteria* was the predominant phylum in the heavy DNA of the PCB group, suggesting that *Cyanobacteria* are involved in PCB-driven BNF.

### Potential polychlorinated biphenyl-metabolizing functions of the active diazotrophic community

Tax4Fun2 was used to predict the functional potential of the active diazotrophic community. A total of 44 PCB metabolism-related functional genes were predicted, including the biphenyl degradation pathway genes *bphA* (K18088), *bphB* (K08690), and *bphC* (K00462) and the benzoate degradation pathway genes (Table S5). The abundances of these predicted functional genes in the heavy fraction of the active diazotrophic community are shown in Fig. S4.

**Figure 4:**
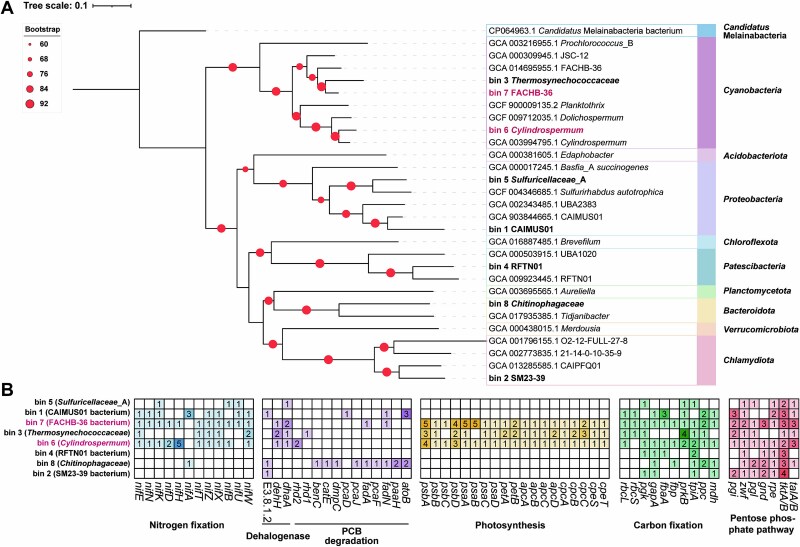
Phylogeny and key metabolic potential of the microbial community represented by heavy fraction genomes. (A) Phylogenetic tree of heavy fraction genomes. Branches with bootstrap values >60 are indicated. (B) Key metabolic potential of the microbial community represented by heavy fraction genomes. The numbers in the small boxes represent the number of functional genes annotated. *rhd1*: PPDO and related ring-hydroxylating dioxygenase gene. *rhd2*: ring-hydroxylating dioxygenase gene. *dhaA*: haloalkane dehalogenase [EC:3.8.1.5] gene. *dehH*: haloacetate dehalogenase [EC:3.8.1.3] gene. *rhd*1 and *rhd*2 are used only to distinguish different dioxygenase genes and do not refer to specific gene names. Details of the remaining abbreviations are provided in Table S5.

Furthermore, metagenome-binning analysis of the heavy DNA from the PCB group yielded 8 high-quality MAGs (Fig. 4A**,**  Table S6) affiliated with *Bacteroidota* (1 MAG), *Chlamydiota* (1 MAG), *Cyanobacteria* (3 MAGs), *Patescibacteria* (1 MAG), and *Proteobacteria* (2 MAGs). The cyanobacterial MAGs were associated with *Thermosynechococcaceae* (bin 3), *Cylindrospermum* (bin 6), and FACHB-36 (bin 7). The functional annotation of the three cyanobacterial MAGs (Fig. 4B) revealed that bins 6 and 7 harbored important genes involved in BNF, including nitrogenase iron protein (*nifH*), molybdenum-iron protein (*nifDKN*), molybdenum cofactor biosynthesis protein (*nifE*), and *nif*-specific proteins (*nifXZT*). Additionally, these bins contained key genes involved in the metabolism of halogenated hydrocarbons, such as haloalkanoic acid dehalogenase (*dehH*), haloalkane dehalogenase (*dhaA*) and ring-hydroxylating dioxygenase (*rhd*). BLASTP analyses revealed the presence of genes encoding proteins related to PCB degradation in the three cyanobacterial MAGs (Tables S7–S9), including the presence of an amino acid sequence in bin 6 with 62.61% similarity to the amino acid sequence encoded by the *bphA* gene of the cyanobacterium *Microcystis aeruginosa* NIES-2520 (Table S8). These results suggest potential for both BNF and PCB metabolism. In addition, all three cyanobacterial MAGs contained essential genes related to photosynthesis and the oxidative pentose pathway (OPP), indicating a mixotrophic lifestyle (Fig. 4B).

### Polychlorinated biphenyl metabolism–nitrogen fixation bifunctionality of *Cylindrospermum* sp.

Based on the metagenome-binning results and functional predictions of the heavy DNA, genomes of three *Cylindrospermum* species, which belong to the same genus as bin 6, were selected for functional annotation analysis. All genomes include nitrogen-fixation genes, and *Cylindrospermum* sp. NIES-4074 also harbors *dehH*, *dhaA*, and *rhd* genes (Fig. S5). Moreover, similar to the results of the genomic annotation of the three *Cyanobacteria* MAGs described above, functional genes related to photosynthesis and OPP were found in the three *Cylindrospermum* genomes. Degradation gene mining also revealed the presence of amino acid sequences similar to those encoded by *bphA*, *bphB* and *bphC* (Table S10).

**Figure 5:**
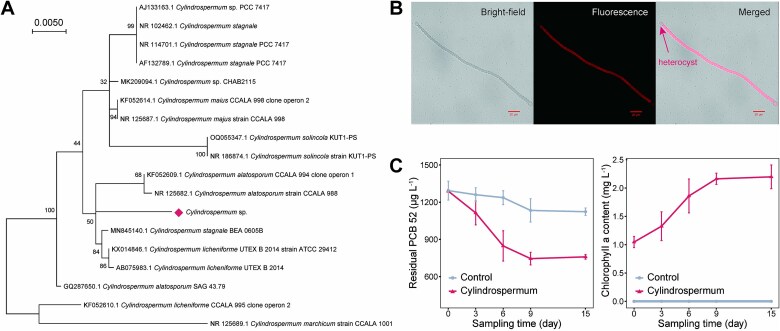
Microbial morphology and characteristics of *Cylindrospermum* sp. (A) Phylogenetic tree of *Cylindrospermum* sp. based on 16S rRNA gene sequence homology. The tree was constructed with MEGA X 10.2.6 software using the maximum likelihood method with the HKY + G + I model. The value on each branch indicates the bootstrap value. The microorganisms indicated by asterisks are the strains used in this study. (B) Cell morphology and arrangement observed under a confocal scanning laser microscope with a 40× objective lens. Bar = 20 μm. Bright-field images (left), images of autofluorescence at a laser wavelength of 561 nm (middle), and merged images (right) are shown. (C) PCB52 degradation curves and growth curve of *Cylindrospermum* sp. Mean values are shown; vertical lines indicate s.d. Control: negative control with no culture present; *Cylindrospermum*: treatment with *Cylindrospermum* sp. culture addition.

Validation experiments confirmed the nitrogen-fixing and PCB52-degrading capabilities of the *Cylindrospermum* sp. Taxonomic identification of the strain based on 16S rRNA gene phylogeny revealed a close affiliation with *Cylindrospermum stagnale* BEA 0605B (Fig. 5A). The nitrogenase activity of this heterocyst-forming filamentous cyanobacterium (Fig. 5B) was 0.959 ± 0.051 nmol C_2_H_2_ mL^−1^ h^−1^ and 16.993 ± 0.905 nmol C_2_H_2_ min^−1^ mg^−1^ chlorophyll. Finally, the strain achieved 42.27% degradation of PCB52 after 9 days in medium with PCB52 as the sole carbon source and nitrogen from air as the sole nitrogen source (Fig. 5C). Two PCB degradation products, 2-chlorobiphenyl and 2,5-dihydroxybenzonic acid, were detected after incubation (Fig. S6).

## Discussion

Diazotroph-driven BNF is one of the most important biogeochemical processes in soil, especially in nitrogen-limited organic-polluted soils. In this study, we identified the active diazotrophic community in PCB-contaminated paddy soil using DNA-SIP and evaluated the potential metabolic pathways of this community through metagenome binning. Finally, we selected a representative microbial strain for functional validation, which revealed the multiple ecological roles diazotrophs may play in PCB metabolism. The results of this study provide a new perspective on the bioremediation of organic-contaminated paddy soils.

Organic pollutants affect soil microbial community composition and ecological functions [48, 49]. BNF is an energy-intensive process that is sensitive to the availability of carbon and nitrogen sources [9, 48]. We previously demonstrated that long-term PCB contamination significantly increases diazotroph abundance and nitrogenase activity in paddy soil [34]. In the present study, we observed similar responses to short-term PCB exposure, perhaps due to similarities in physicochemical properties and microbial community structure between the soil used in this study and the soil used in our previous study (Fig. 1B and C). Other organic pollutants have similar effects on the soil microbial community; for example, oil pollution increased the gene copy number and gene expression of *nifH* in sediments by 60% and 5-fold [50].

Multiple lines of evidence from this study indicate the involvement of PCB degraders in the enhancement of PCB52 degradation by nitrogen fixation: (i) PCB increased nitrogenase activity, diazotroph abundance, and PCB degradation gene abundance, especially during the mid-to-late stage of the incubation period (Figs 1B and C, and 2A); (ii) nitrogenase activity had a significant negative linear correlation with the residual PCB52 concentration in the soil (Fig. 1D); and (iii) the abundance of diazotroph genes increased synchronously with the increase in the abundance of PCB degradation genes during the incubation period (Fig. 2C). A previous study showed that adding molybdate to soil to stimulate the nitrogenase activity of indigenous microorganisms increased the degradation rate of PAHs in agricultural soil by 1.06-fold [15]. The results of the present study confirm that diazotrophs play an important role in pollutant metabolism by facilitating the transformation of organic pollutants through a variety of mechanisms, including nitrogen supply and biodegradation [4, 48].

In long-term organic-polluted soils, microorganisms adapted to the environment gradually emerge. In our previous study, the PCB-responsive diazotrophic group included *Bradyrhizobium*, *Desulfomonile*, and *Cyanobacteria*, which formed the core diazotrophic ecological cluster and were the primary drivers of BNF in PCB-contaminated soil [34]. Interestingly, *Bradyrhizobium*, *Desulfobacterota*, and *Cyanobacteria* were also identified as active diazotrophs in the PCB-contaminated paddy soil in this study (Fig. S2). Certain diazotrophic genera within these groups have been reported to possess PCB degradation capabilities [51–53]. Other active PCB-degrading microorganisms that have been identified in soil include nitrogen fixers such as *Azoarcus*, *Azoarcus* sp. T, *Azospirillum*, *Azotobacter vinelandii*, *Burkholderia* and *Nostoc punciforme* (a member of Cyanobacteria) [23, 53, 54]. Among these microorganisms, *Burkholderia* and *Nostoc* were identified as active diazotrophs in the present study (Table S3). These bifunctional microorganisms kill two birds with one stone: they degrade pollutants while providing nutrients to other degraders through nitrogen fixation, further promoting pollutant degradation [16].

Cyanobacteria are the primary source of non-symbiotic nitrogen fixation and can contribute 10–80 kg N ha^−1^ to wetland rice ecosystems [26, 55]. Cyanobacteria are the predominant active diazotrophs in rice–soil systems [26] and were the dominant active diazotrophic taxon in the PCB-contaminated paddy soil in this study (Figs 3B and S3). Taken together with the presence of nitrogen-fixing cyanobacterial aggregates in oil-contaminated sediments, our results suggest that *Cyanobacteria* well adapt organic-contaminated environments [56, 57]. Although cyanobacterial bin 3 was annotated with *nifW*, *nifX*, and *nifZ*, auxiliary protein genes that are essential for nitrogenase synthesis [28], it lacked the *nifH* structural nitrogenase gene, possibly due to incomplete genome recovery.

**Figure 6:**
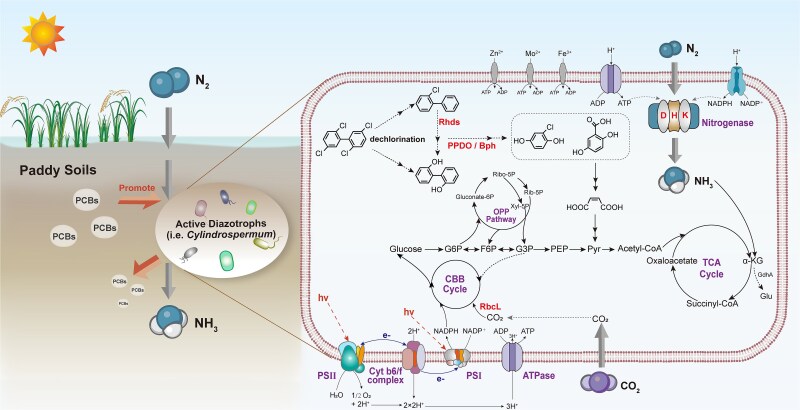
Metabolic pathways in mixotrophic cyanobacteria genomes predicted to mediate PCB metabolism. Rhds: ring-hydroxylating dioxygenase. Bph: biphenyl dioxygenase. PPDO: phenylpropionate dioxygenase. RbcL: ribulose-bisphosphate carboxylase. OPP pathway: oxidative pentose pathway. CBB cycle: Calvin–Benson–Bassham cycle. TCA cycle: tricarboxylic acid cycle. Ribo-5P: ribulose-5-phosphate. Rib-5P: ribose-5-phosphate. Xyl-5P: xylulose-5-phosphate. hν: photon energy. G6P: glucose-6-phosphate. F6P: fructose-6-phosphate. G3P: glyceraldehyde-3-phosphate. PEP: phosphoenolpyruvate. Pyr: pyruvate. α-KG: α-ketoglutarate. GdhA: glutamate dehydrogenase. PSII: Photosystem II. Cyt b6/f complex: cytochrome b6/f complex. PSI: photosystem I. ATPase: F-type ATP synthase.

Cyanobacteria can degrade various persistent organic pollutants, including phenols, pesticides, PAHs, and PCBs [51, 58, 59]. Our findings further confirm the potential dual capabilities of mixotrophic cyanobacteria for N_2_ fixation and utilization of organic pollutants as carbon sources (Fig. 6). From the perspective of the reaction process, biological nitrogen fixation and PCB degradation are coupled. Biological nitrogen fixation is a high-energy-consuming process [9], and mixotrophic cyanobacteria that can utilize PCB as a carbon source can obtain energy from PCB metabolism to further promote biological nitrogen fixation. At the same time, hydrogen ions produced by biological nitrogen fixation can serve as electrons for PCB metabolism [22, 23]. At the genetic level, the genome of *Cylindrospermum* sp. contains genes that can participate in PCB degradation. One of the cyanobacteria in this study (bin 6) harbored a gene encoding ring-hydroxylating dioxygenase (RHD), which is involved in PCB and biphenyl degradation [60, 61]. The other cyanobacteria (bin 3) contained a phenylpropionate dioxygenase (PPDO) gene that is very similar to *bphA1* [62, 63]. Genes similar to PCB-degrading functional genes were present in all three mixotrophic cyanobacteria (Tables S7–S9). Additionally, all three *Cyanobacteria* were annotated with genes encoding haloacetate dehalogenase (*dehH* [EC:3.8.1.3]) and haloalkane dehalogenase (*dhaA* [EC:3.8.1.5]), hydrolytic dehalogenases that are involved in the dehalogenation of various halogenated compounds, such as haloalkanes, cycloalkanes, and alkenes [64, 65]. These dehalogenases may participate in downstream PCB degradation pathways starting from chlorobenzoates and chloro-2-hydroxy-2,4-pentadienoates [3]. From the perspective of metabolic products, the presence of 2-chlorobiphenyl and 2,5-dihydroxybenzoic acid indicates that PCB has undergone dechlorination and degradation. Previous study has reported that *Anabaena* PD-1 anaerobically dechlorinates PCBs via 3-chlorobenzoate-3,4-dioxygenase [66], so it cannot be ruled out that new PCB dechlorination genes exist in *Cylindrospermum* sp.

Heterocyst-forming cyanobacteria are pivotal in maintaining and enhancing soil fertility in paddy fields [28, 67]. The representative active diazotrophic strain *Cylindrospermum* sp. used in this study is a heterocyst-forming filamentous cyanobacterium (Fig. 5B), consistent with the morphological classification of *Cyanobacteria* [27]. Through a spatial separation mechanism involving heterocysts, Cyanobacteria can perform nitrogen fixation under aerobic conditions by using energy from photosynthesis, thus elegantly resolving the "oxygen paradox" [28]. This mechanism suggests the coexistence of aerobic and microoxic cellular environments within heterocyst-forming cyanobacteria. Similar to the mechanism by which symbiotic nitrogen fixation drives PCB transformation [22], autotrophic nitrogen-fixing cyanobacteria may drive PCB transformation in the presence of a low-oxygen nitrogen-fixing environment and the nitrogen-fixing product hydrogen. The unique co-existence of microoxic and aerobic environments within heterocyst-forming cyanobacteria and the molecular size and hydrophobicity of PCB allow PCB metabolism to be "relayed" between heterocysts and ordinary cells. Consequently, heterocyst-forming cyanobacteria may be an ideal natural environment for the complete mineralization of highly chlorinated PCBs. This speculation is supported to some extent by the formation of both dechlorination and oxidative degradation products of PCB52 by *Cylindrospermum* sp. Unlike most PCB-metabolizing bacteria, versatile mixotrophic cyanobacteria may be more readily adapt to the unique "flooding-drying" state of the soil environment during the rice growth cycle. This adaptation provides new options for the bioremediation of PCB-contaminated paddy soil and is of great significance for the future development of environmentally friendly management strategies centered on the "3B" technology: bioremediation, biological nitrogen fixation, and biological carbon fixation.

## Conclusions

This study utilized ^15^N-DNA-SIP and metagenomic binning techniques to identify active nitrogen-fixing microbial taxa in PCB-contaminated paddy soil and assess their PCB-metabolizing potential. The identified active diazotrophs were distributed across 14 phyla, predominantly *Cyanobacteria*. Mixotrophic cyanobacteria, particularly genera such as FACHB-36 and *Cylindrospermum*, exhibited potential for nitrogen fixation, pollutant metabolism, and photosynthesis. Metabolic product analysis indicated that both dechlorination and degradation metabolic pathways are present in some *Cyanobacteria* (i.e. *Cylindrospermum*). These findings provide new insights into the pollutant-metabolizing potential of active diazotrophs, especially mixotrophic cyanobacteria, in PCB-contaminated paddy soil. The dual roles of diazotrophs in nitrogen fixation and pollutant transformation could be harnessed to develop more effective bioremediation strategies for organic-contaminated soils.

## Supplementary Material

3_Supporting_information_20241211_plain_text_ycae160

4_Supporting_Table_20241211_ycae160

## Data Availability

Raw sequences data of llumina Miseq sequencing are accessible from the National Center for Biotechnology Information (BioProject PRJNA1122340 and PRJNA1122350, Sequence Read Archive accession numbers SRR29354318-SRR29354323 and SRR29354292-SRR29354294).
