# Supplementary material for: Mixotrophic cyanobacteria are critical active diazotrophs in polychlorinated biphenyl-contaminated paddy soil
Source: ISME Commun. 2025 Mar 18;5(1):ycae160. doi: 10.1093/ismeco/ycae160 (PMC11924043; doi:10.1093/ismeco/ycae160)
Supplement: 3_Supporting_information_20241211_plain_text_ycae160 [file 3_supporting_information_20241211_plain_text_ycae160.docx]

*Supplementary information for*

**Mixotrophic cyanobacteria are critical active diazotrophs in polychlorinated biphenyl-contaminated paddy soil**

Wenbo Hu ^a, b, c^, Ying Teng ^a, c *^, Xiaomi Wang ^a, c^, Yongfeng Xu ^a, c^, Yi Sun ^a, b, c^, Hongzhe Wang ^a, b^^, c^, Yanning Li ^a, b, c^, Shixiang Dai ^a, c^, Ming Zhong ^a^, Yongming Luo ^a, c^

*^a^ Key Laboratory of Soil and Sustainable Agriculture, Institute of Soil Science, Chinese Academy of Sciences, Nanjing 211135, China*

*^b^ University of Chinese Academy of Sciences, Beijing 100049, China*

*^c^ University of Chinese Academy of Sciences, Nanjing 211135, China*

^*^ Corresponding authors.

Key Laboratory of Soil and Sustainable Agriculture

Institute of Soil Science

Chinese Academy of Sciences

No. 298 Chuangyou Road, Jiangning District, Nanjing, Jiangsu Province 211135, China

Tel: +86-025-86881531

E-mail address: [yteng@issas.ac.cn](mailto:yteng@issas.ac.cn)

**List of** **Supplemental files**

Supplementary information (**Figures S1-S6; Tables S1-S10; Supplementary Text S1-S9**) accompanies this paper.

**Fig. S1** The schematic diagram of the experimental workflow in this study.

**Fig. S2** Phylogenetic tree of 16S rRNA genes showing the sequence diversity and taxonomic distribution of active diazotrophs.

**Fig. S3** Relative abundances (%) of *Cyanobacteria* in heavy DNA fractions from ^15^N microcosms and ^14^N microcosms.

**Fig. S4** Functional gene prediction for biphenyl degradation pathways and benzoate degradation pathways in active diazotrophs based on Tax4Fun.

**Fig. S5** Key metabolic potential of the microbial communities in *Cylindrospermum* genomes.

**Fig. S6** Mass spectra of the identified metabolites during PCB52 degradation in culture of *Cylindrospermum* sp.

**Table S1.** Soil physio-chemical properties.

**Table S2**. Primers and amplification conditions of qPCR analyses.

**Table S3.** Relative abundances (%) of active diazotrophs in heavy DNA fractions from ^15^N-PCB treatment and ^14^N-PCB treatment.

**Table S4.** *Cylindrospermum* genomes found in NCBI GenBank.

**Table S5.** The information of functional genes.

**Table S6.** Taxonomic information of high-quality MAGs.

**Table S7.** The characters of genes involved in PCBs-degradation calculated by BLASTP in the genome of bin 3.

**Table S8.** The characters of genes involved in PCBs-degradation calculated by BLASTP in the genome of bin 6.

**Table S9.** The characters of genes involved in PCBs-degradation calculated by BLASTP in the genome of bin 7.

**Table S10.** The characters of genes involved in PCBs-degradation calculated by BLASTP in the three genomes of *Cylindrospermum* species.

**Text S1.** Measurement of soil physicochemical properties.

**Text S2.** Sampling for soil microcosm incubation.

**Text S3.** Reasons for primers selection

**Text S4.** Purification of DNA fraction.

**Text S5.** Details of the assembly, binning, taxonomic classification and functional annotation of metagenomic sequencing reads**.**

**Text S6.** The BG11 medium composition.

**Text S7.** Determination the taxonomic information of *Cylindrospermum* sp.

**Text S8.** Microscopy and determination nitrogenase activity of *Cylindrospermum* sp.

**Text S9.** Extraction and analysis of chlorophyll a, PCB 52, and degradation metabolic intermediates.


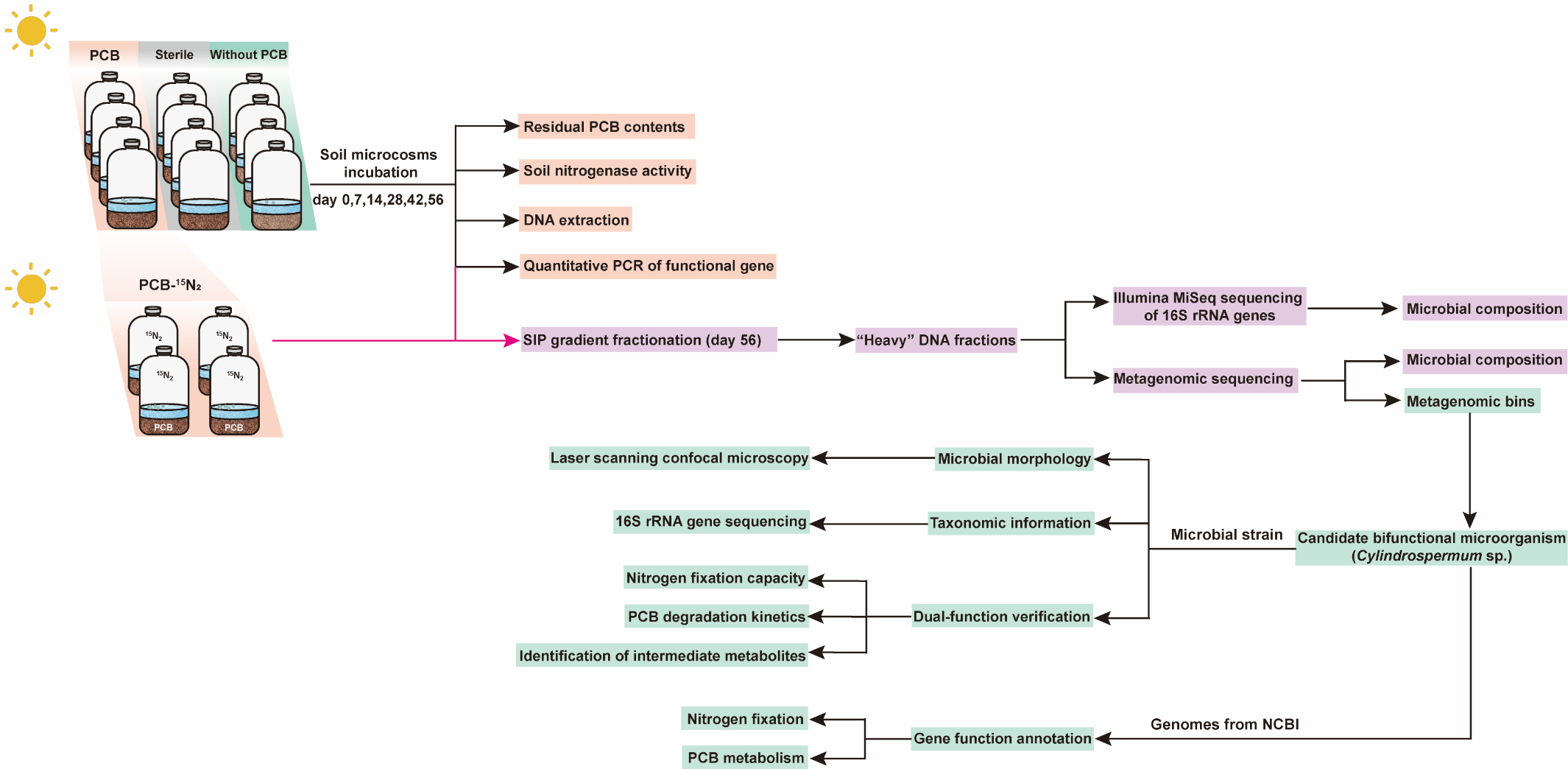


**Fig. S1** The schematic diagram of the experimental workflow in this study.

**
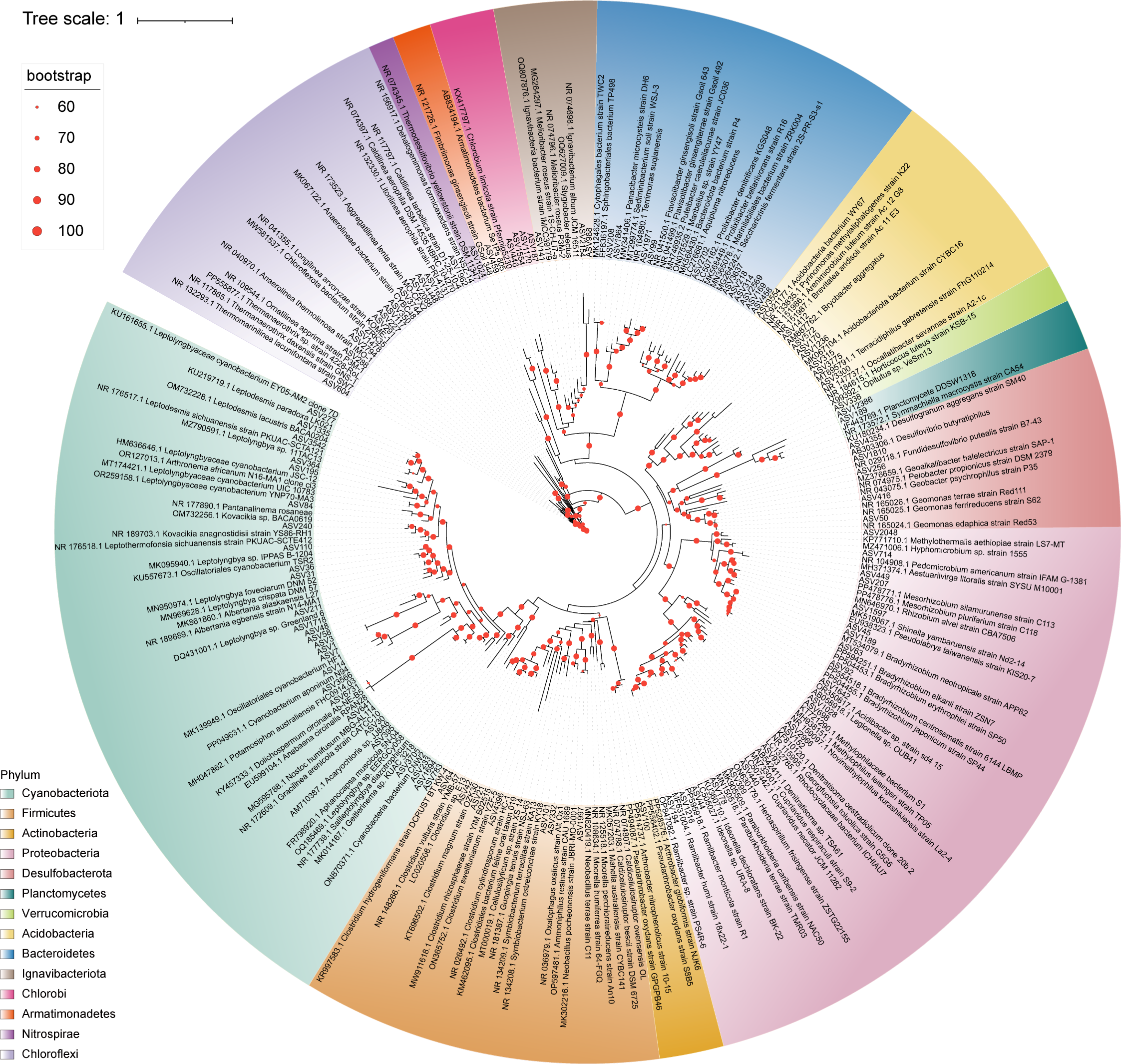
**

**Fig. S2** Phylogenetic tree of 16S rRNA genes showing the sequence diversity and taxonomic distribution of active diazotrophs. The leaves are colored at the phylum level. Branches with bootstrap values greater than 60 are marked with red dots.

**
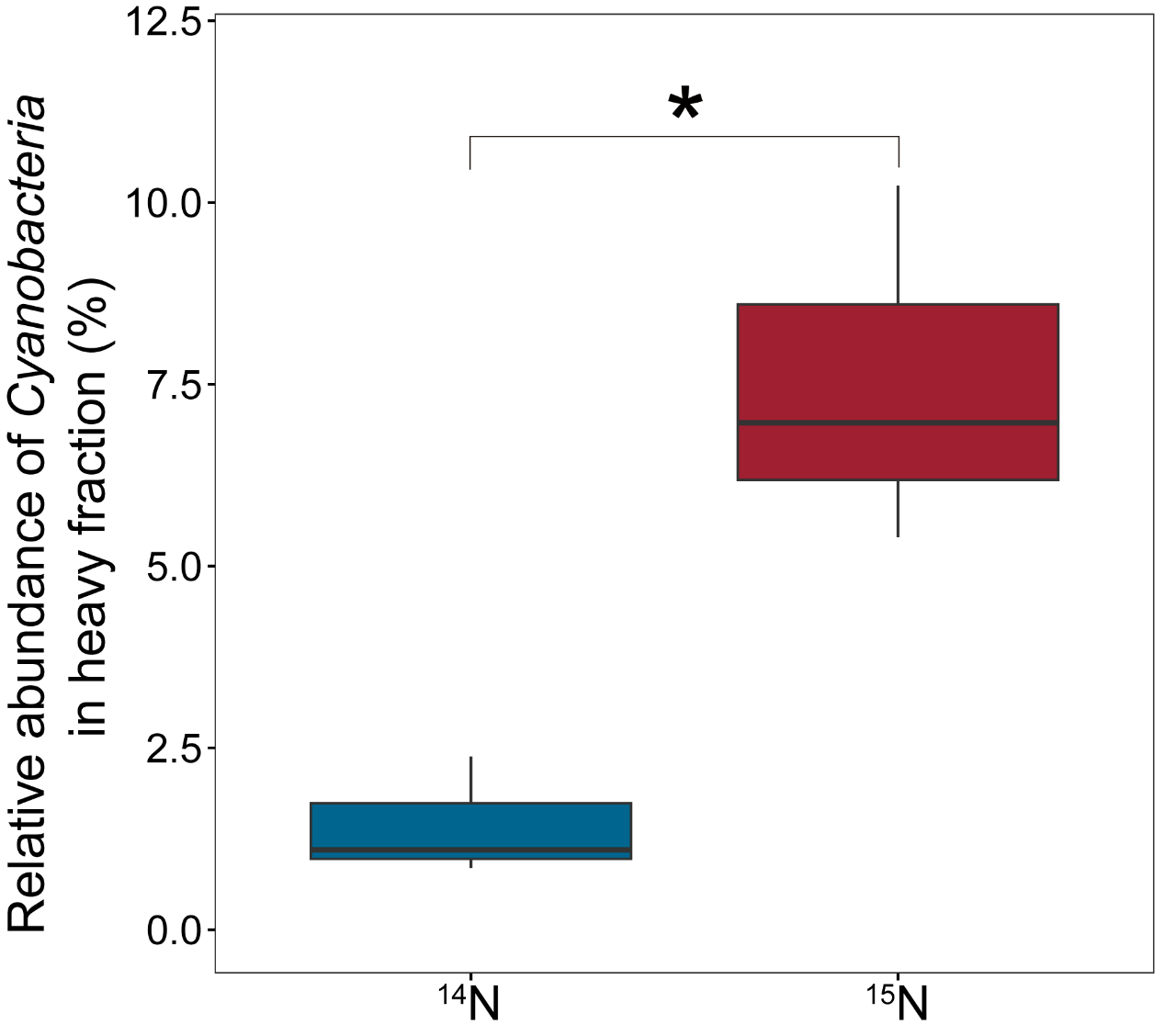
**

**Fig. S3** Relative abundances (%) of *Cyanobacteria* in heavy DNA fractions from ^15^N microcosms and ^14^N microcosms. * indicates *P* < 0.05.

**
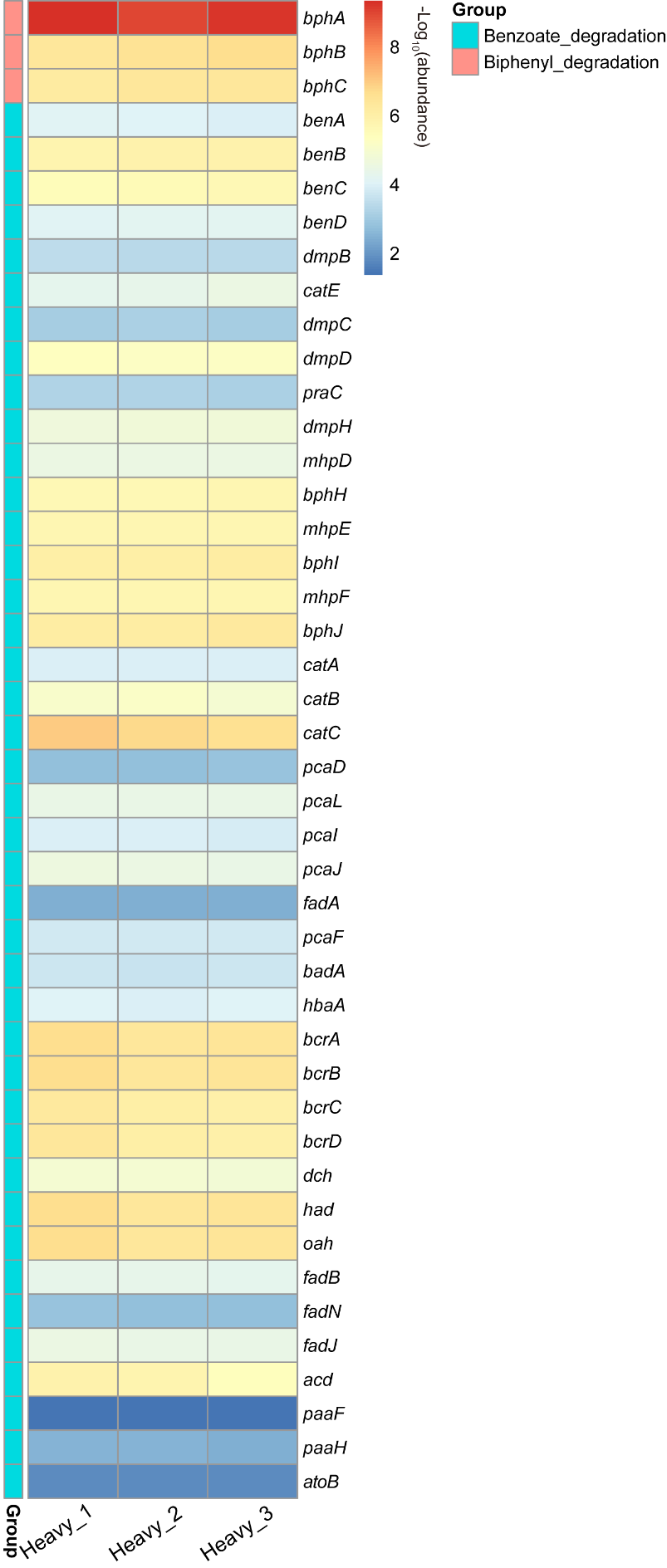
**

**Fig. S4** Functional gene prediction for biphenyl degradation pathways and benzoate degradation pathways in active diazotrophs based on Tax4Fun.


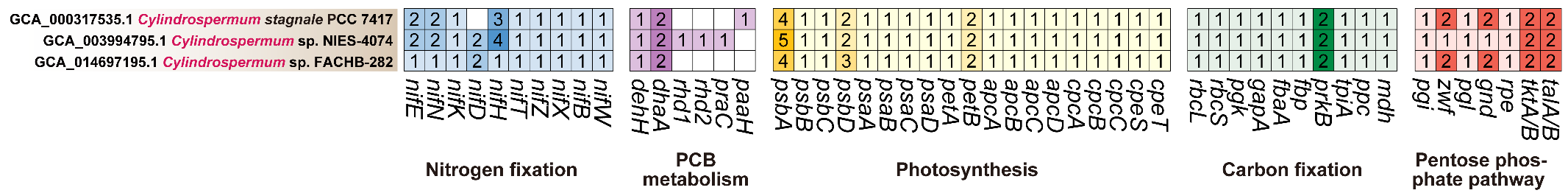


**Fig. S5** Key metabolic potential of the microbial communities in *Cylindrospermum* genomes. The numbers in the small boxes represent the number of functional genes annotated. *rhd1*: phenylpropionate dioxygenase (PPDO) and related ring-hydroxylating dioxygenase. *rhd2*: ring-hydroxylating dioxygenase. *dhaA*: haloalkane dehalogenase [EC:3.8.1.5]. *dehH*: haloacetate dehalogenase [EC:3.8.1.3]. Details of the remaining abbreviations are provided in Table S5.

**
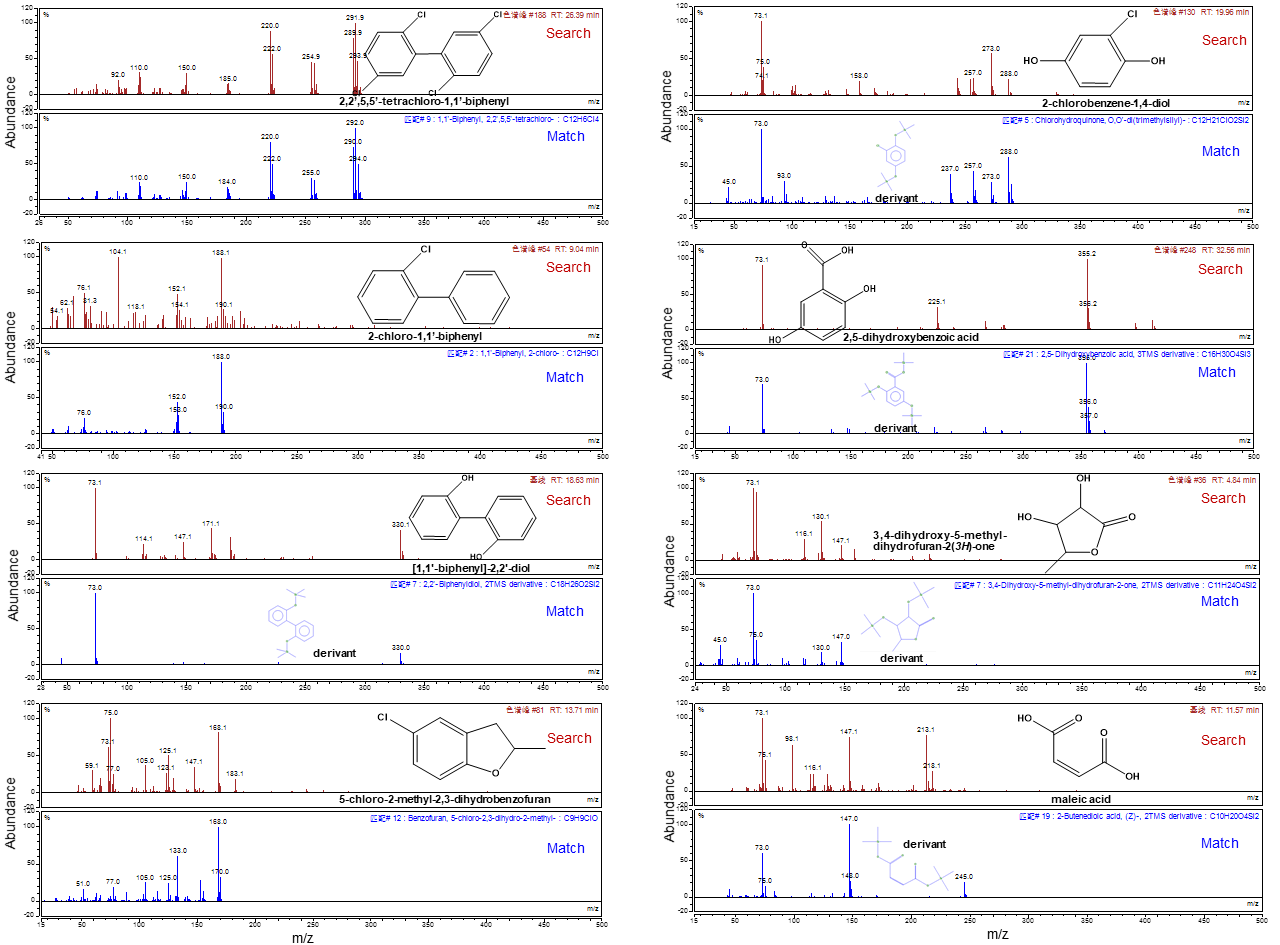
**

**Fig. S6** Mass spectra of the identified metabolites during PCB52 degradation in culture of *Cylindrospermum* sp.

**Table S1** Soil physio-chemical properties

| Parameters (units) | Soil |
| --- | --- |
| pH | 6.19 ± 0.01 |
| Organic matter (g kg^-1^) | 60.38 ± 0.11 |
| Total nitrogen (g kg^-1^) | 0.35 ± 0.04 |
| Ammonium nitrogen (mg kg^-1^) | 7.96 ± 0.05 |
| Nitrate nitrogen (mg kg^-1^) | 0.17 ± 0.29 |
| PCB52 (μg kg^-1^) | ND^*^ |

* ND = Not detected.

**Table S2** Primers and amplification conditions of qPCR analyses^*^

| **Target** | **Primer name** | **Primer sequence (5’–3’)** | **Thermal profile for qPCR** | **Reference** |
| --- | --- | --- | --- | --- |
| 16S rRNA genes | 515F | GTGCCAGCMGCCGCGG | initial denaturation 95℃ 5 min; 35 cycles of 94℃ 30 s, 55℃ 30 s, 72℃ 30 s; extension 72℃ 5 min | [1] |
|  | 907R | CCGTCAATTCMTTTRAGTTT |  |  |
| *nifH* | polF | TGCGAYCCSAARGCBGACTC | initial denaturation 95℃ 10 min, 40 cycles of 95℃ 30 s, 55℃ 30 s, 72℃ 30 s; extension 72℃ 5 min | [2] |
|  | polR | ATSGCCATCATYTCRCCGGA |  |  |
| *bphC3* (gene for PCB degrading bacteria) | bphC-q3-188f | CAGGCTTGGAAGTGGATGACG | initial denaturation 94℃ 5 min, 35 cycles of 94℃ 45 s, 58℃ 45 s, 72℃ 40 s; extension 72℃ 10 min | [3] |
|  | bphC-q3-333r | GCCGAACGGATCTTGCAGAC |  |  |
| *Dehalococcoides* (*Dhc*) 16S rRNA genes (gene for PCB dechlorinating bacteria) | C-DehalF | CGCTTTAAGTGTCCCGCC | initial denaturation 94℃ 5 min, 35 cycles of 94℃ 45 s, 58℃ 45 s, 72℃ 40 s; extension 72℃ 10 min | [3] |
|  | C-1100R | GGGTTGCGCTCGTTG |  |  |

* Reasons for primers selection in **Text S3**.

**Text S1. Measurement of soil physicochemical properties**

Soil physicochemical properties were analyzed with the previous methods [4]. Briefly, Soil pH was determined using a pH meter (PB-21, Sartorius, Germany). Soil TN was measured by Kjeldahl digestion (KJELTEC 8420, FOSS, Denmark). NH_4_^+^-N and NO_3_^-^-N were measured by using a continuous flow analytical system (San++ system, SAKLAR, Netherlands) after extraction with 2 mol L^−1^ KCl. SOM was determined using the K_2_Cr_2_O_7_–H_2_SO_4_ oxidation method. A parallel sample was examined every 10 samples, and the relative deviation was 93.2%–102.8%.

**Text S2. Sampling for soil microcosm incubation**

For the soil microcosm cultivation experiment, we set up four biological replicates at each time point for each treatment group, with 10 g of soil in each culture bottle, and destructive sampling was conducted on days 0, 7, 14, 28, 42, and 56. The specific sampling procedure was as follows: first, we used the acetylene reduction assay (ARA) method [5] to measure nitrogenase activity. Subsequently, we removed the aluminum foil cover and butyl rubber stopper, wrapped the bottle mouth with parchment paper, and placed the culture bottles into a pre-freezing chamber at -25°C for pre-freezing. After pre-freezing, the samples underwent freeze-drying under vacuum for 48h. Finally, all samples were thoroughly mixed. A portion of the samples was quickly stored at -80°C for DNA extraction, and the remaining samples were ground through a 60-mesh sieve for PCB52 content determination.

**Text S3.** **Reasons for primers selection**

Microorganisms primarily metabolize PCBs via anaerobic reductive and aerobic oxidative pathways [6]. When considering the quantification of PCB-metabolizing microorganisms, we reviewed a significant amount of literature and ultimately decided to use the *bphC3* and *Dehalococcoides* (*Dhc*) 16S rRNA gene from Chen et al. [3]. The reasons are as follows:

1. The tested soil used in this study and in the referenced study are both paddy soils from Taizhou, Zhejiang Province.
2. The referenced study reported a wide range of Bph primers, including *bphA1-A9* and *bphC1-C5*. After trying all the primers, we found that, consistent with the results in the referenced study, only *bphC3* was present in substantial quantities in the tested soil. Therefore, we chose *bphC3* as the functional gene for quantifying aerobic degraders.
3. There remain limited reports on specific genes responsible for anaerobic dechlorination of PCBs [7]. The C-DehalF/C-1100R primers targets microbial groups capable of participating in PCB dechlorination, such as *Dehalococcoides* and o-17/DF-1. This pair of primers was designed and modified based on probes that selectively detect microorganisms involved in reductive dechlorination of PCBs [8]. We relied on the *Dhc* 16S rRNA gene used in the referenced study as the gene for quantifying anaerobic dechlorinators.

**Text S4. Purification of DNA fraction**

The DNA purification were performed as described by Neufeld et al. [9]. Briefly, 550 µl PEG6000 and 0.5 µL Glycogen (20 mg/mL; Roche, Basel, Switzerland) were added to each DNA fraction, mixed and heated at 37℃ for 1 h for DNA precipitation. Then centrifuge at 20°C at 13,000 ×g for 30 min and remove the supernatant. Then 500 µl of 70% ethanol was added to wash the DNA precipitate, inverted to mix well and continued centrifugation for 10 min to remove the supernatant. Repeat the previous cleaning step, blow-dry the residual alcohol in a sterile ultra-clean bench and then dissolve the DNA with 20 µL of TE buffer (1.0M, pH = 8.0), store at -20℃.

**Text S5. Details of the assembly, binning, taxonomic classification and functional annotation of metagenomic sequencing reads**

Details of the assembly, binning, taxonomic classification and functional annotation of raw reads can refer to Dong et al [10]. In brief, raw reads were filtered by the metaWRAP Read_QC module (parameters: -skipbmtagger) to generate clean reads by removing adaptor sequences, trimming, and removing low-quality reads. Then, the assembly module of metaWRAP (parameters: -megahit) for quality-controlled reads was individually assembled and co-assembled. Short contigs (<1000 bp) were removed, followed by strain-level cluster analysis and redundancy removal were carried out with dRep (parameters: -comp 50 -con 10) to obtain metagenome-assembled genomes (MAGs). CheckM then was utilized to evaluate the completeness and contamination of MAGs [11]. The MAGs with completion > 50% and contamination < 10% **(Table S4)** were subjected to taxonomy classification using GTDB-TK, as well as genes annotation using MetaERG against the KEGG and EggNOG database [12, 13]. Detailed information on functional genes related to PCBs metabolic pathways and nitrogen fixation pathway are described in the **Table S5**.

To construct a phylogenetic tree with reference genomes, we initially selected reference genomes from a pre-built genome tree available in the Genome Taxonomy Database (GTDB) [12]. The query genomes were subsequently placed onto this reference framework using the phylogenetic placement tool, pplacer [14]. This approach enabled us to identify the most closely related genomes, which were then chosen as reference genomes for further analysis. Combining the results of Hug et al. [15] and Coleman et al. [16], we selected *Candidatus* Melainabacteria bacterium (CP064963.1) as an outgroup to root the phylogenetic tree. The UBCG pipeline [17] was applied to construct the phylogenetic tree, ensuring a robust and comprehensive analysis. The resulting tree was visualized using the Interactive Tree of Life (iTOL) platform [18], facilitating a detailed examination and presentation of the phylogenetic relationships.

**Text S6. The BG11 medium composition**

The modified nitrogen-free BG11 medium [19] contained the following components per liter: 0.04 g of K_2_HPO_4_, 0.075 g of MgSO_4_ 7H_2_O, 0.036 g of CaCl_2_ 2H_2_O, 6.0 mg of citric acid, 6.0 mg of ferric ammonium citrate, 1.0 mg of Na_2_EDTA, 0.02 g of Na_2_CO_3_, and 1.0 mL of trace element solution A5. The trace element solution A5 contained (L^−1^): 2.86 g of H_3_BO_3_, 1.81 g of MnCl_2_ 4H_2_O, 0.222 g of ZnSO_4_ 7H_2_O, 0.39 g of Na_2_MoO_4_ 2H_2_O, 0.079 g of CuSO_4_ 5H_2_O, and 51.13 mg of CoSO_4_ 7H_2_O.

**Text S7. Determination the taxonomic information of *Cylindrospermum* sp.**

Genomic DNA was extracted from log-phase bacterial cultures grown in BG11 medium using a bacterial genomic DNA extraction kit (TaKaRa, Tokyo, Japan). The DNA concentration and quality were assessed using a Nanodrop ND-1000 spectrophotometer. The 16S rRNA gene was amplified using the primers 27F (AGAGTTTGATCCTGGCTCAG) and 1492R (TACGACTTAACCCCAATCGC) [20]. PCR amplification products were purified with DNA Gel Extraction kit (Vazyme, Nanjing, China) followed by Sanger sequencing. The resulting DNA sequences were identified using the NCBI Blastn online tool (https://blast.ncbi.nlm.nih.gov/Blast.cgi). Reference sequences were retrieved from the NCBI GenBank database, and a phylogenetic tree was constructed using MEGA X 10.2.6 with MUSCLE for sequence alignment and the maximum likelihood method with the HKY+G+I model [21, 22].

*DNA sequence information obtained from sequencing:*

>Cylindrospermum_16S_1395bp

CGGGGGGGTGCTTACACATGCAAGTCGAACGGTCTCTTCGGAGATAGTGGCGGACGGGTGAGTAACGCGTGAGAATCTACCTTCAGGTCTGGGACAACCACTGGAAACGGTGGCTAATACCGGATGTGCCGAGAGGTGAAAGGTTAACTGCCTGAAGAAGAGCTCGCGTCTGATTAGCTTGTTGGTGGGGTAAAAGCCTACCAAGGCGACGATCAGTAGCTGGTCTGAGAGGATGATCAGCCACACTGGGACTGAGACACGGCCCAGACTCCTACGGGAGGCAGCAGTGGGGAATTTTCCGCAATGGGCGAAAGCCTGACGGAGCAATACCGCGTGAGGGAGGAAGGCTCTTGGGTTGTAAACCTCTTTTCTCAGGGAATAAAAAAATGAAGGTACCTGAGGAATAAGCATCGGCTAACTCCGTGCCAGCAGCCGCGGTAATACGGAGGATGCAAGCGTTATCCGGAATGATTGGGCGTAAAGCGTCCGCAGGTGGCTATGTAAGTCTGCTGTTAAAGAGCAAGGCTCAACCTTGTAAAGGCAGTGGAAACTACATAGCTAGAGTGCGTTCGGGGCAGAGGGAATTCCTGGTGTAGCGGTGAAATGCGTAGATATCAGGAAGAACACCAGTGGCGAAAGCGCTCTGCTAGGCCGCAACTGACACTGAGGGACGAAAGCTAGGGGAGCGAATGGGATTAGATACCCCAGTAGTCCTAGCCGTAAACGATGGATACTAGGCGTTGCTTGTATCGACCCGAGCAGTGCCGTAGCTAACGCGTTAAGTATCCCGCCTGGGGAGTACGCACGCAAGTGTGAAACTCAAAGGAATTGACGGGGGCCCGCACAAGCGGTGGAGTATGTGGTTTAATTCGATGCAACGCGAAGAACCTTACCAAGGCTTGACATGTCGCGAATCCCTCTGAAAGGAGGGAGTGCCTTCGGGAGCGCGAACACAGGTGGTGCATGGCTGTCGTCAGCTCGTGTCGTGAGATGTTGGGTTAAGTCCCGCAACGAGCGCAACCCTCGTTTTTAGTTGCCAGCACTTCGGGTGGGCACTCTAGAGAGACTGCCGGTGACAAACCGGAGGAAGGTGGGGATGACGTCAAGTCAGCATGCCCCTTACGCCTTGGGCTACACACGTACTACAATGCTACGGACAGAGGGCAGCAAGCATGCGAGTGCAAGCTAATCCCGGAAACCGTAGCTCAGTTCAGATCGCAGGCTGCAACTCGCCTGCGTGAAGGAGGAATCGCTAGTAATTGCAGGTCAGCATACTGCAGTGAATTCGTTCCCGGGCCTTGTACACACCGCCCGTCACACCATGGAAGCTGGTCACGCCCGAAGTCGTTACCCCAACCTTTGGAGGGGGATGCCTAAGCAGGACCTTGGC

**Text S8. Microscopy and** **determination nitrogenase activity of *Cylindrospermum* sp.**

The logarithmic phase of the bacterial suspension was mixed well, and 5 μL of the suspension was added dropwise to the slide with a sterile gun tip and covered with a coverslip. The morphology and arrangement of cells were observed by confocal scanning laser microscopy (CLSM, LSM710, Carl Zeiss, Tena, Germany) under a 40x objective, and autofluorescence was observed under excitation at 561 nm.

Nitrogenase activity of logarithmic stage strains was assayed by acetylene reduction method [5, 23]. *Cylindrospermum* sp. was cultured to logarithmic stage (OD680=0.38) with BG11 medium, and 5 mL was taken in sterile 20 mL culture flasks, which were sealed with butyl rubber stoppers and aluminium foil lids, and then 10% of the air above was replaced with high-purity acetylene. The ethylene content in the gas above was detected by a gas chromatograph (Agilent Technologies, Santa Clara, CA) after 24 h of incubation. The specific detection method was the same as that of soil nitrogenase activity analysis.

**Text S9. Extraction and analysis of chlorophyll a, PCB 52, and degradation metabolic intermediates**

A modified hot ethanol extraction method was used to determine the chlorophyll a content in the bacterial suspension [24, 25]. The cultured 5mL bacterial suspension was sonicated for 30min (25℃, 100% frequency), 10mL anhydrous ethanol was added, and maintained in a water bath at 80℃ for 30 min, and then left for 6h at room temperature away from light. The absorbance of the extract at 750, 663, 645 and 630 nm was detected by MQX200 microplate reader (Bio-Tek, Inc, Winooskie, Vermont, USA). The chlorophyll a content was calculated using the following equation: *Chla* (μg mL^-1^) = 11.64×(*OD*_663_-*OD*_750_)-2.16×(*OD*_645_-*OD*_750_)+0.10×(*OD*_630_-*OD*_750_). Where *Chla* is the concentration of chlorophyll a (μg mL ^-1^), and *OD*_663_, *OD*_750_, *OD*_645_, *OD*_630_ are the absorption values at wavelengths 662, 750, 645 and 630nm, respectively.

The extraction and detection methods for PCB52 and its degradation metabolites were adapted from the previously described protocol [26]. 5 mL of dichloromethane (HPLC grade; TEDIA, Ohio, USA) were added to the culture bottles, mixed thoroughly, and sonicated for 30 min (25℃, 100% frequency). Subsequently, 5 mL of n-hexane was added, mixed well, and sonicated again. After phase separation, the upper organic layer was collected. A portion was filtered through a 0.22 μm organic membrane into an amber vial and stored at -20℃ for GC analysis of PCB52 content. The remaining extract was evaporated to dryness under a gentle nitrogen stream, followed by the addition of 0.1 mL n-hexane and 0.1 mL derivatization reagent (N,O-bis(trimethylsilyl)acetamide/TMS-B; assay > 95%). The mixture was incubated at 60℃ for 30 min, diluted with n-hexane to 1 mL, filtered through a 0.22 μm organic membrane into an amber vial, and subjected to GC-MS for qualitative analysis of degradation metabolites.

PCB 52 and degradation metabolism intermediates were analyzed by GC and GC-MS, respectively. A GC7890 gas chromatograph (Agilent Technologies, Santa Clara, CA) was used to analyze PCB 52 content. The detector was μ-ECD, the column was HP-5 (30 m × 0.32 mm × 0.25 μm), the carrier gas was high-purity nitrogen (60 mL min^-1^), and the injection volume was 1.0 μL. The temperatures of the inlet port and the detector were 270℃ and 300℃, respectively. The column warming procedure was as follows: first to 180℃, then to 210℃ at a rate of 30℃ min^-1^, followed by 4℃ min^-1^ to 240℃, and finally to 280℃ at 30℃ min^-1^ and held for 1 min. The quantification of PCB 52 was performed using the external standard method (standard curve *R*^2^ = 0.9996). Spiking recovery experiments determined that PCB 52 recoveries ranged from 84% to 108%. Degradation metabolites were qualitatively analyzed using a gas chromatograph-mass spectrometer Trace1300 ISQ7000 (Thermo Fisher Scientific, Waltham, USA) equipped with a TG-5SILMS capillary column (30 m × 0.25 mm × 0.25 μm). Ultra-high purity nitrogen was used as the carrier gas (1.0 mL min^-1^), and the injection volume was 1.0 μL in splitless mode. The inlet temperature was 250℃. The oven temperature program was as follows: initial temperature 80℃ held for 2 min, then ramped at 5℃ min^-1^ to 300℃ and held for 5 min. The EI ion source temperature was set at 280℃, and the mass range scanned was 45-500 amu.

**References:**

1. Turne S, Pryer KM, Miao VPW, Palmer JD. Investigating Deep Phylogenetic Relationships among Cyanobacteria and Plastids by Small Subunit rRNA Sequence Analysis1. Journal of Eukaryotic Microbiology. 1999; 46:327-38.

2. Poly F, Monrozier LJ, Bally R. Improvement in the RFLP procedure for studying the diversity of *nifH* genes in communities of nitrogen fixers in soil. Res Microbiol. 2001; 152:95-103.

3. Chen C, Yu C, Shen C, Tang X, Qin Z, Yang K, et al. Paddy field – a natural sequential anaerobic–aerobic bioreactor for polychlorinated biphenyls transformation. Environ Pollut. 2014; 190:43-50.

4. Xu Y, Teng Y, Wang X, Ren W, Zhao L, Luo Y et al. Endogenous biohydrogen from a rhizobium-legume association drives microbial biodegradation of polychlorinated biphenyl in contaminated soil. Environ Int. 2023; 176:107962.

5. Hardy RWF, Holsten RD, Jackson EK, Burns RC. The acetylene-ethylene assay for N_2_ fixation: laboratory and field evaluation. Plant Physiol. 1968; 43:1185-207.

6. Passatore L, Rossetti S, Juwarkar AA, Massacci A. Phytoremediation and bioremediation of polychlorinated biphenyls (PCBs): state of knowledge and research perspectives. J Hazard Mater. 2014; 278:189-202.

7. Wang S, Chng KR, Wilm A, Zhao S, Yang K-L, Nagarajan N, et al. Genomic characterization of three unique *Dehalococcoides* that respire on persistent polychlorinated biphenyls. P Natl Acad Sci. 2014; 111:12103-08.

8. Sowers K, Fagervoid S, Watts J, May H Gene Probes for the selective detection of microorganisms that reductively dechlorinate polychlorinated biphenyl compounds. Google Patents.

9. Neufeld JD, Vohra J, Dumont MG, Lueders T, Manefield M, Friedrich MW, et al. DNA stable-isotope probing. Nature Protocols. 2007; 2:860-66.

10. Dong X, Zhang C, Peng Y, Zhang H, Shi L, Wei G, et al. Phylogenetically and catabolically diverse diazotrophs reside in deep-sea cold seep sediments. Nat Commun. 2022; 13:4885.

11. Parks DH, Imelfort M, Skennerton CT, Hugenholtz P, Tyson GW. CheckM: assessing the quality of microbial genomes recovered from isolates, single cells, and metagenomes. Genome Res. 2015; 25:1043-55.

12. Chaumeil P-A, Mussig AJ, Hugenholtz P, Parks DH. GTDB-Tk: a toolkit to classify genomes with the Genome Taxonomy Database. Bioinformatics. 2019; 36:1925-27.

13. Dong X, Strous M. An integrated pipeline for annotation and visualization of metagenomic contigs. Front Genet. 2019; 10.

14. Matsen FA, Kodner RB, Armbrust EV. pplacer: linear time maximum-likelihood and Bayesian phylogenetic placement of sequences onto a fixed reference tree. BMC Bioinformatics. 2010; 11:538.

15. Hug LA, Baker BJ, Anantharaman K, Brown CT, Probst AJ, Castelle CJ, et al. A new view of the tree of life. Nat Microbiol. 2016; 1:16048.

16. Coleman GA, Davín AA, Mahendrarajah TA, Szánthó LL, Spang A, Hugenholtz P, et al. A rooted phylogeny resolves early bacterial evolution. Science. 2021; 372:eabe0511.

17. Na S-I, Kim YO, Yoon S-H, Ha S-m, Baek I, Chun J. UBCG: Up-to-date bacterial core gene set and pipeline for phylogenomic tree reconstruction. J Microbiol. 2018; 56:280-85.

18. Letunic I, Bork P. Interactive Tree Of Life (iTOL) v5: an online tool for phylogenetic tree display and annotation. Nucleic Acids Res. 2021; 49:W293-W96.

19. Rippka R, Deruelles J, Waterbury JB, Herdman M, Stanier RY. Generic assignments, strain histories and properties of pure cultures of cyanobacteria. Microbiology. 1979; 111:1-61.

20. Frank JA, Reich CI, Sharma S, Weisbaum JS, Wilson BA, Olsen GJ. Critical Evaluation of Two Primers Commonly Used for Amplification of Bacterial 16S rRNA Genes. Appl Environ Microb. 2008; 74:2461-70.

21. Edgar RC. MUSCLE: a multiple sequence alignment method with reduced time and space complexity. BMC Bioinf. 2004; 5:113.

22. Kumar S, Stecher G, Li M, Knyaz C, Tamura K. MEGA X: Molecular Evolutionary Genetics Analysis across Computing Platforms. Mol Biol Evol. 2018; 35:1547-49.

23. Hu W, Wang X, Wang X, Xu Y, Li R, Zhao L, et al. Enhancement of nitrogen fixation and diazotrophs by long-term polychlorinated biphenyl contamination in paddy soil. J Hazard Mater. 2023; 446:130697.

24. Zhang H, Jiang X, Lu L, Xiao W. Biodegradation of polychlorinated biphenyls (PCBs) by the novel identified cyanobacterium *Anabaena* PD-1. PLoS One. 2015; 10:e0131450.

25. Mackinney G. Absorption of light by chlorophyll solutions. J biol chem. 1941; 140:315-22.

26. Wang X, Teng Y, Tu C, Luo Y, Greening C, Zhang N, et al. Coupling between nitrogen fixation and tetrachlorobiphenyl cechlorination in a rhizobium–legume symbiosis. Environ Sci Technol. 2018; 52:2217-24.
